# CAREGIVER EXPERIENCE OF TELEHEALTH-DELIVERED HOME SAFETY EVALUATIONS

**DOI:** 10.1093/geroni/igz038.912

**Published:** 2019-11-08

**Authors:** Megan Gately

**Affiliations:** Bedford VA Medical Center Geriatric Research, Education & Clinical Center, Bedford, Massachusetts, United States

## Abstract

People with dementia are living in the community, necessitating in-home supports for their day-to-day needs. Given geriatrics work force shortages, innovative strategies that increase the reach of extant providers while maintaining quality are needed. Home-based video telehealth may increase access to specialty care such as a dementia-focused home safety evaluation by an occupational therapist; however, little is known about the technological demands and caregiver experience of a home safety evaluation delivered by telehealth. Our study employed video telehealth to deliver a dementia-focused home safety evaluation compared to in-person evaluation for caregivers (n=10) of veterans with dementia. Most video visits encountered technological problems. Caregiver experience between the video and in-person evaluations differed. Our findings reflect the highly dynamic, complex nature of in-home video telehealth which requires maximal collaboration with caregivers. By explicating the resource demands and potential burden of video telehealth for caregivers, development of effective in-home telehealth evaluation is enhanced

